# Acute Exhaustive Exercise under Normoxic and Normobaric Hypoxic Conditions Differentially Regulates Angiogenic Biomarkers in Humans

**DOI:** 10.3390/medicina57070727

**Published:** 2021-07-19

**Authors:** Frank Suhr, Sarah Knuth, Silvia Achtzehn, Joachim Mester, Markus de Marees

**Affiliations:** 1 Exercise Physiology Research Group, Department of Movement Sciences, Biomedical Sciences Group, KU Leuven, 3000 Leuven, Belgium; 2 The German Research Center of Elite Sport, German Sport University Cologne, 50933 Cologne, Germany; sarah.dorsch@gmx.net (S.K.); achtzehn@dshs-koeln.de (S.A.); joachim.mester@t-online.de (J.M.); 3 Institute of Cardiology and Sports Medicine, German Sport University Cologne, 50933 Cologne, Germany; 4 Department of Sports Medicine and Sports Nutrition, Ruhr-University Bochum, 44801 Bochum, Germany; markus.demaress@rub.de

**Keywords:** hypoxia, VO_2_max, endostatin, MMPs, angiogenic modulators

## Abstract

*Background and Objectives:* Angiogenesis describes the outgrowth of new capillaries from already existing ones. Different biomarkers regulate this process. Physical exercise and hypoxia are key stimuli for the activation of different angiogenic molecules, such as the vascular endothelial growth factor (VEGF). matrix metalloproteases (MMPs)-2 and -9 or the extracellular matrix cleavage fragment endostatin. The present study aimed to investigate influences of short-term, intensive cycling exercise under both normoxic and normobaric hypoxic conditions on the mentioned parameters. *Materials and Methods:* Twelve male subjects (age: 23.3 ± 2.0 years) participated in the study. All subjects conducted four intensive cycling tests until individual exhaustion in a randomized order under the following conditions: normoxia, 2000 m, 3000 m and 4000 m above sea level. Blood samples were taken before (pre) and 10 min, 30 min, 60 min and 240 min post exercise and were analyzed by ELISA. *Results:* VEGF showed a significantly reduced concentration compared to the pre-value solely under 4000 m at 10 min post exercise. MMP-2 showed significantly reduced concentrations at 240 min post exercise under 4000 m. MMP-9 increased at 240 min post exercise under both 2000 m and 4000 m conditions. Endostatin was significantly increased at 10 min post exercise independently of the applied stimulus. *Conclusions:* The presented data show that intensive short-term exercise bouts facilitate the bioavailability of angiogenic, ECM (extracellular matrix)-related biomarkers. This finding is interesting for both health- and performance-related research as it demonstrates the positive effects of intensive short exercise interventions.

## 1. Introduction

Physical exercise performance depends on the skeletal muscle tissue oxygen and nutrient support [1]. Consequently, the peripheral capillary bed plays a crucial role in the regulation of exercise capacities. The peripheral capillary bed possesses the capacity to increase in density through a process called angiogenesis, which characterizes the outgrowth of new capillaries for already existing ones [2]. This would result in a higher capillary-to-skeletal muscle fiber ratio with the consequence of enhanced endurance performance [3]. Furthermore, the acute regulation of the vascular permeability supports oxygen and nutrient support during acute exercise [3]. Finally, the acute adaptation of the vascular diameter through endothelial cell-derived nitric oxide (NO^•^) is an important mechanism to supply working tissues, e.g., skeletal muscles under acute exercise [4].

A variety of blood-detectable biomarkers contributes to the regulation of the mentioned processes. Of note, the vascular endothelial growth factors (VEGF) is the most potent one to induce angiogenesis and vascular permeability [5]. Both processes receive support by the matrix metalloproteases (MMP), specifically MMP-2 and MMP-9, known as gelatinases [6]. The main functions of MMP-2 and MMP-9 in the periphery are the liberation of the growth factors stored in the extracellular matrix (ECM) [7,8]. This remodeling process involves the generation of ECM-derived cleavage fragments with biological properties [7]. One important cleavage fragment is endostatin, the NC1 domain-specific fragment of collagen XVIII, which is an endothelial cell-specific collagen [9]. Endostatin is actively involved in the acute control of the vascular diameter as it induces the generation of NO^•^ by activating the endothelial nitric oxide synthase (eNOS) [4]. Hence, VEGF, MMP-2, MMP-9 and endostatin are interesting biomarkers, whose acute regulations could give insights into performance-supporting mechanisms.

Our group demonstrated that both different exercise interventions [10,11] as well as changes in environmental conditions [10] challenge angiogenic modulators. However, it remains unclear, whether acute bouts of exhaustive exercise, as applied during athlete testing and in regular training cycles, change VEGF, MMP-2, MMP-9 and endostatin concentrations in the blood stream. To shed light on this interesting exercise physiology-related question, we subjected healthy young participants to a series of acute bouts of exhaustive exercise at normoxic (sea level), simulated hypoxia of 2000 m, 3000 m and 4000 m above sea level in a randomized order. We took blood samples at different time points at assess the concentrations of VEGF, MMP-2, MMP-9 and endostatin.

## 2. Materials and Methods

### 2.1. Subjects

Twelve male subjects (age: 23.3 ± 2.0 years, height: 181.1 ± 6.2 cm, body weight: 74.9 ± 8.7 kg, rel. VO_2_max: 56.7 ± 7.7 mL·kg^−1^·min^−1^; training hours/week: 10.3 ± 3.7 h) participated in this study. All participants abstained from alcohol consumption 24 h prior to and during the training intervention, were non-smokers and did not take medication regularly. No respiratory and cardiac abnormalities were determined in the pre-study medical examinations and none of the subjects reported of chronic diseases. All test persons were informed about the study contents and gave their written consent for participation in the examinations, diagnoses and blood drawings. The study protocol was approved by the ethical committee of the German Sport University Cologne and is in line with the Declaration of Helsinki.

### 2.2. Study Design

Each participant completed four cycling tests in a randomized order at intervals of one week on a SRM cycle ergometer (Schoberer, Jülich, Germany) under different conditions: (a) normal ambient conditions (sea levels); (b) three different normobaric hypoxic conditions (2000 m (FiO_2_ = 16.3%), 3000 m (FiO_2_ = 14.2%), 4000 m (FiO_2_ = 12.7%), respectively). Normobaric hypoxic conditions were generated using a hypoxic chamber (Hypoxico Inc., New York, USA). The VO_2_max, the maximal power output (P_max_) and the arterial oxygen saturation (SaO_2_) were measured during exercise.

### 2.3. Exercise Protocol

**Figure 1:**
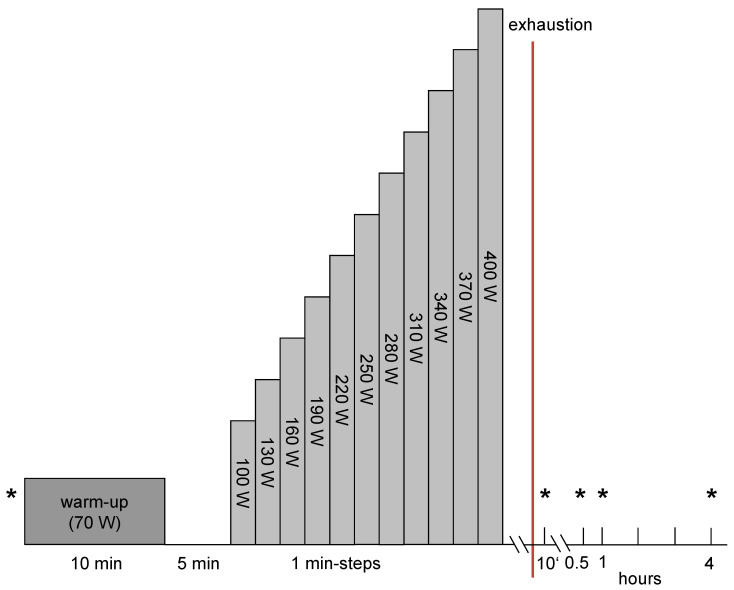
Exercise protocol. * = venous blood samples.

The VO_2_max test was held to an incremental exercise protocol (Figure 1). After taking the first venous blood samples the athletes carried out a 10 min warm-up program with an output of 70 Watts (W) with arbitrary pedaling frequency.

Starting with 100 W the output was increased every minute by 30 W until individual exhaustion. The exercise was aborted when the subjects were unable to perpetuate the given pedaling frequency of 70–80 round per minute (rpm) or if the test was discontinued due to individual exhaustion. The measurement of the spirometry data (MetaLyzer 3B, Cortex) began with the start of the incremental exercise. During all exercise procedures under the different ambient conditions, the SaO_2_ was measured (C3 Patients monitor, Philips GmbH, Hamburg, Germany) to determine the impact/severity of exercise and the respective simulated altitude.

### 2.4. Measurement of VEGF, MMP-2, MMP-9 and Endostatin Serum Levels

Venous blood samples were drawn at the following time points: pre-exercise, 10 min (min) post, 30 min post, 60 min post and 240 min post exercise (Figure 1). 9.5 mL of blood was collected with the EDTA containing Vacutainer^®^ blood withdrawal system (Becton Dickinson, Franklin Lakes, NJ, USA). After storage at 7 °C for approximately 30 min, after which blood coagulation was finished, the blood samples were centrifuged for 10 min at 1861× *g* and 4° C (Rotixa 50, Hettich Zentrifugen, Mühlheim, Germany). The serum was stored at −80° C. Serum levels of VEGF (pg·mL^−1^), MMP-2 (ng·mL^−1^), MMP-9 (ng·mL^−1^) and endostatin (ng·mL^−1^) were determined by using human ELISA kits (R&D Systems, Wiesbaden, Germany).

### 2.5. Statistics

All data are listed as mean ± standard deviation. The statistical analyses were carried out by the use of STATISTICA for Windows, Version 7.1 (Statsoft, Tulsa, OK, USA). Statistical prerequisites (normal distribution, homogeneity of variance) were fulfilled. Dependent t-tests were applied to check significance between two test points. Differences between the four conditions were analyzed via analysis of variance (ANOVA) with repeated measurements and a Tukey post-hoc test. The level of significance was set at *p* < 0.05.

## 3. Results

### 3.1. P_max_ and Relative VO_2_max

The mean maximal power (P_max_) decreased significantly during the three VO_2_max tests under hypoxic conditions compared to sea level values. The power declined at maximal workload from 367 ± 48 W (sea level) to 342 ± 42 W at 2000 m (−6.7%, *p* < 0.001), 325 ± 39 W at 3000 m (−11.4%, *p* < 0.001) and to 308 ± 41 W at an altitude of 4000 m (−16.1%, *p* < 0.001).

The mean values for the relative VO_2_max of the twelve subjects under sea level was 56.7 ± 7.7 mL·kg^−1^·min^−1^. As expected, during all tests under hypoxic conditions (2000 m: −14.8%, *p* < 0.001; 3000 m: −21.2% *p* < 0.001 and 4000 m: −24.9% *p* < 0.001) the VO_2_max decreased significantly compared to the pre-values at sea level.

### 3.2. SaO_2_

We observed a significant decrease of the SaO_2_ at maximal workload during all tests under hypoxic conditions compared to NN values. The values decreased from 94.5 ± 4.7% at sea level to 84.2 ± 7.2% at 2000 m (−10.3%, *p* < 0.001), 78.1 ± 7.8% at 3000 m (−16.4%, *p* < 0.001) and to 71.8 ± 8.7% at 4000 m (−22.7%, *p* < 0.001).

### 3.3. VEGF

**Figure 2:**
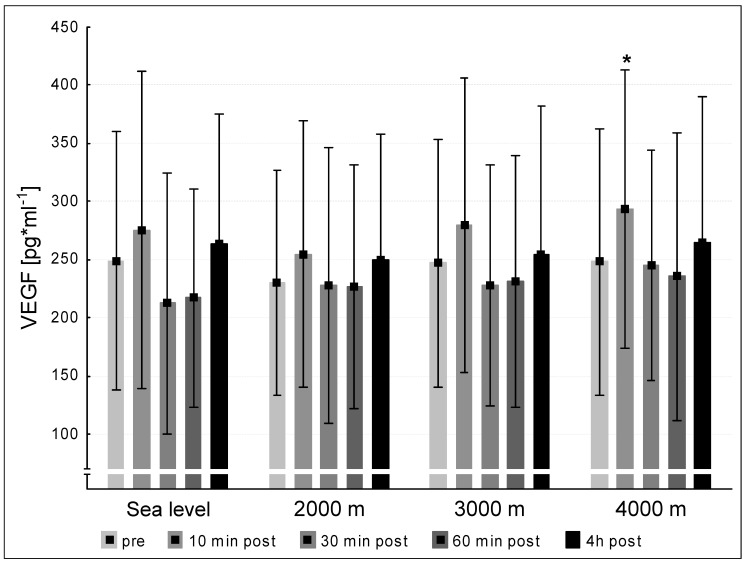
VEGF (vascular endothelial growth factor) concentration at the five measurement time points (pre, 10 min post, 30 min post, 60 min post, 240 min post exercise) during the different ambient conditions (sea level, 2000 m, 3000 m, 4000 m). Mean ± standard deviation. * = *p* < 0.05 to pre.

The mean pre-values of VEGF of the twelve subjects did not differ significantly at the four testing conditions sea level, 2000 m, 3000 m and 4000 m. Solely at an altitude of 4000 m the VEGF level significantly increased at 10 min post exercise compared to the pre-value (<0.05, Figure 2).

### 3.4. MMP-2 and -9

**Figure 3:**
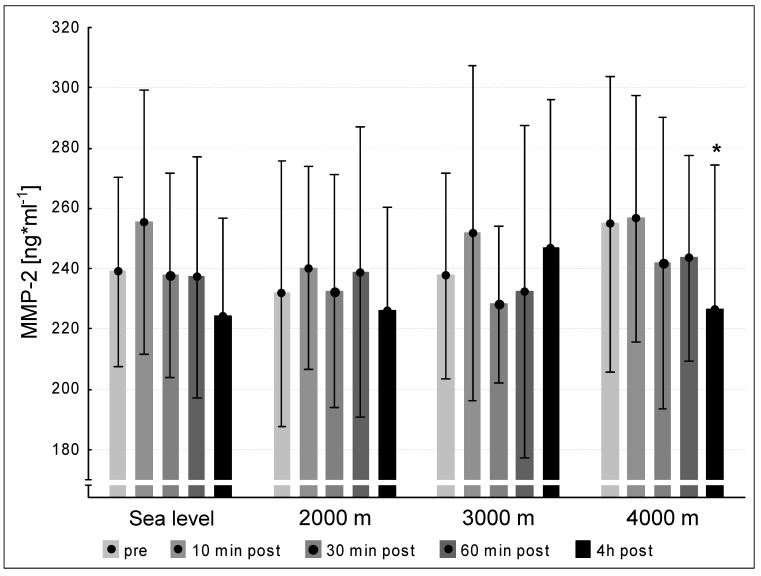
MMP-2 concentration at the five measurement time points (pre, 10 min post, 30 min post, 60 min post, 240 min post exercise) during the different ambient conditions (sea level, 2000 m, 3000 m, 4000 m). Mean ± standard deviation. * *p* < 0.05 to pre.

**Figure 4:**
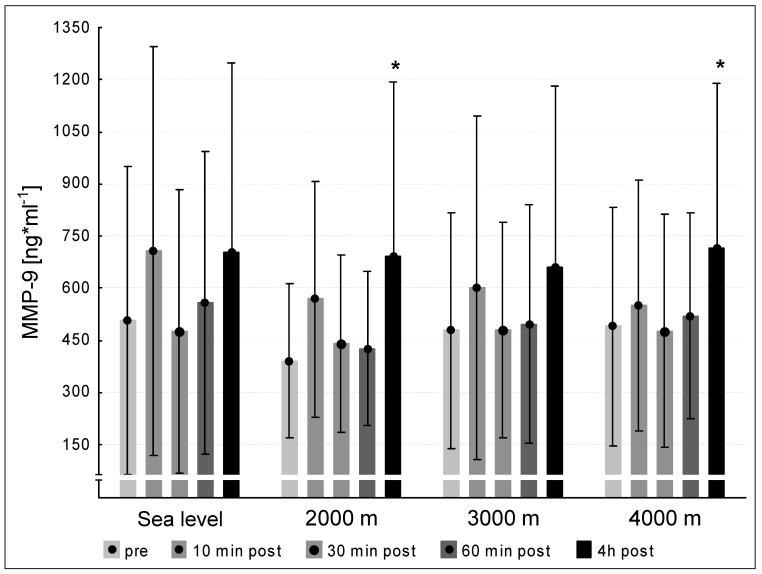
MMP-9 concentration at the five measurement time points (pre, 10 min post, 30 min post, 60 min post, 240 min post exercise) during the different ambient conditions (sea level, 2000 m, 3000 m, 4000 m). Mean ± standard deviation. * *p* < 0.05 to pre.

We did not observe significant differences between the pre concentrations of MMP-2 at sea level and the hypoxic interventions. MMP-2 values at 240 min post exercise decreased significantly at 4000 m compared to pre-exercise (*p* < 0.05, Figure 3). We found significant increases between the pre-values of MMP-9 at sea level and the three altitude conditions. At 2000 m and 4000 m, the MMP-9-concentrations increased significantly at 240 min post exercise (*p* < 0.05, respectively). At 3000 m MMP-9 showed a tendency to increase (*p* = 0.051, Figure 4).

### 3.5. Endostatin

**Figure 5:**
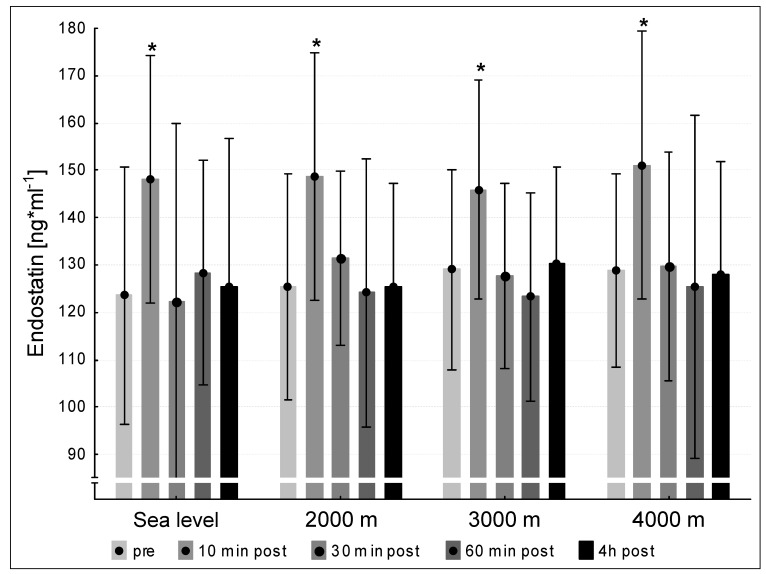
Endostatin concentration at the five measurement time points (pre, 10 min post, 30 min post, 60 min post, 240 min post exercise) during the different ambient conditions (sea level, 2000 m, 3000 m, 4000 m). Mean ± standard deviation. * *p* < 0.05 to pre.

We did not find significant differences between the mean pre-values of endostatin at the four testing conditions sea level, 2000 m, 3000 m and 4000 m. Endostatin concentrations increased significantly 10 min post exercise after each interventions (*p* < 0.05 for all hypoxic conditions) compared to pre-values (Figure 5). At the three following points of measurement (30 min post, 60 min post, 240 min post exercise) the endostatin values declined to pre-level under all tested conditions sea level, 2000 m, 3000 m and 4000 m (Figure 5).

## 4. Discussion

Here, we demonstrate that regulations of exercise capacity-related angiogenic markers occur within a very short period upon acute exhaustion exercise under sea level as well as under the tested hypoxic conditions. Specifically, VEGF increased hypoxia-dependent at 10 min post exercise at 4000 m. MMP-2 did not show any acute response within a period of four hours post exercise under any tested conditions, whereas MMP-9 increased at four hours post-acute exhaustive exercise under 2000 m and 4000 m. Finally, the ECM-derived cleavage fragment endostatin increased at 10 min upon acute exhaustive exercise under each tested condition. These data demonstrate that acute exhaustive exercise initiates fast responses at the cytokine level to react to extreme changes provoked by such stimulations.

VEGF is one of the most potent angiogenic growth factor [12]. VEGF increases upon exercise [10,13,14]. Both mechanical forces (e.g., exercise) and hypoxia stimulate VEGF production and release. Hypoxia occurs locally in skeletal muscle tissue and can be enhanced by oxygen-reduced ambient conditions [15]. Mechanical stimuli increase as a result of enhanced blood flow during exhaustive exercise and subsequently elevate shear stress at the vessel walls [16,17].

In the present study, we observed a significant increase of the circulating VEGF concentrations at 10 min post exercise compared to the pre-value under hypoxic conditions at 4000 m. It is possible that a combination of exhaustive short-term exercise and severe hypoxic conditions (as reflected by the observed SaO_2_ values) represent a suitable combination to increase VEGF concentrations. The present results show tendencies similar to those found by Kraus et al. [14], who showed an up-regulation of VEGF in plasma directly and 120 min post exercise following a 60 min lasting exercise (50% P_max_). Suhr et al. [10] demonstrated a significant VEGF increase directly after 90 min of cycling exercise and concluded that mechanical rather than hypoxic stimuli control the VEGF increase. The exercise durations used in the studies of Kraus et al. [14] and Suhr et al. [10] lasted considerably longer than in the present study. This implies that the presently applied and discussed hypoxic conditions alone seem to be insufficient to induce releases of VEGF into the circulation (excepted 4000 m), but rather mechanical stimuli and exercise *per se* over a certain period may be responsible for VEGF releases. Finally, the presented data do not represent angiogenic processes per se. However, they demonstrate the potential of acute short-intense exercise to release angiogenesis-triggering molecules, e.g., VEGF, that might in combination with classical long-lasting endurance training result in sufficient angiogenic adaptations.

MMP-2 and MMP-9 contribute to angiogenic events through regulating the balance between pro- and anti-angiogenic factors [7]. MMP-2 and MMP-9 either inhibit angiogenesis by generating angiogenic modulators (e.g., endostatin) [18] or by stimulating angiogenesis by liberation of growth factors (e.g., VEGF) from the extracellular matrix [19]. Whereas MMP-2 appears to be involved in the stabilization and maturing of new capillaries, MMP-9 is attributed to the primary activation and stimulation of endothelial cells and thus the initiating proceedings in the formation of new capillaries [6].

We did not find any increases in MMP-2 levels until 240 min post exercise at any condition. Hence, our intervention was insufficient to increase MMP-2 levels within 240 min. In contrast, we found elevated MMP-9 levels at 240 min post exercise compared to the pre-value under 2000 m and 4000 m conditions. Under 3000 m the MMP-9 level tended to increase (*p* = 0.051). This finding shows that exercise elevates MMP-9 levels to contribute to angiogenic initiations during the first hours of activation.

Several studies showed exercise-induced increase of MMP-2 and MMP-9 [10,20,21]. Our former results [10] are partially in line with the present results as we found increased MMP-2 levels directly after different intensive cycling interventions and an increment of MMP-9 levels 240 min post exercise. Directly after 60 min of intensive uphill treadmill exercise Koskinen et al. [21] found an up-regulation of MMP-2 and MMP-9. The contrary running type—downhill running—increased MMP-9 significantly after a 45 min-lasting exercise bout [20]. The described partial differences between our present study and the discussed ones seem to be caused by different exercise modes and highly divergent exercise durations, which seem to be the major aspect, since in all studies both mechanical and (at least local) hypoxic stimuli were induced. Of note, MMP-9 is directly involved angiogenic remodeling [22]. Of course, the measured circulating MMP-9 levels do not immediately influence the angiogenic phenotype; however, MMP-9 molecules could adhere locally in the peripheral muscle tissue to act as angiogenesis-mediated compounds. The latter event will likely occur upon a long-lasting training intervention, but not after the presented short-intense exercise bout. Finally, it remains speculative until when the MMP-9 levels might remain elevated beyond the 4 h after cessation of exercise as measured in this study. Further studies that evaluate the time course of MMP-9 over a longer period, e.g., 24 h, upon a single short-intense exercise bout could contribute to the clarification of this relevant question.

Initially, endostatin was claimed as a potent angiogenesis inhibitors, as it retarded the VEGF-induced proliferation and migration of endothelial cells as well as enhanced their apoptosis [23,24]. However, recent approaches argue for the function of an angiogenesis modulator with both pro- and anti-angiogenic effects in a concentration-dependent mechanism of action [4,7,25]. However, the exact biological function of endostatin remains unknown [7,26].

Under the four conditions (sea level, 2000 m, 3000 m und 4000 m), endostatin concentrations increased significantly at 10 min post exercise compared to the pre-test and subsequently decreased at 30 min post, 60 min post and at 240 min post exercise to pre-level. Different investigations showed increasing endostatin concentrations after exercise [10,11,13,27]. The present results suggest that the exhaustive exercise tests might have stimulated the protein turnover of the connective tissue and therefore released endostatin as a fragment derived from collagen type XVIII, which is a highly abundant molecule of vascular basement membranes in skeletal muscles [28]. Our group [10] showed that endostatin values increased significantly immediately after 90 min of intensive cycling exercise under both normoxic and hypoxic conditions. In consensus to the present study, we [10] did not observe a potential influence of hypoxic stimuli on the endostatin release. In contrast, Paddenberg et al. [27] found increased endostatins level after a three weeks lasting hypoxic intervention in mice. The protocol of the Paddenberg study was not comparable to the present study with an intervention duration of 10 min and might therefore be responsible for the contrary results. The study of Gu et al. [13] is comparable with the present one regarding the intensity and duration of exercise. The authors found an increase of endostatin after a short-time intensive running exercise (4–10 min) under normoxic conditions. Interestingly, Wenzel and colleagues [4] demonstrated in vitro that endostatin evokes vascular relaxation by increasing nitric oxide demonstrating that endostatin may be a potent regulator of the peripheral vascular tone and may play a key role in the local oxygen supply in the periphery. That implies that physical exercise is an important factor to control peripheral blood flow and vascular tone. Our data reveal that single bouts of exhausting, but short cycling exercise can induce the protein turnover of the ECM independently of external applied hypoxia and therefore contribute to the local supply in the peripheral skeletal system.

Finally, we would like to discuss possible changes of oxidant/antioxidant as well as inflammatory aspects with respect to our study protocol, since these pathways (among others) might influence exercise-induced adaptations. Exercise and training influences both oxidant and antioxidant status of the human body. In this regards, it was demonstrated in rowers that acute exercise bouts elevated protein oxidation status [29]. A similar result was demonstrated in a study, in which professional cyclists performed a single as well as a series of Wingate tests [30]. In contrast, longer period training interventions under hypoxic conditions did not result in changes of protein oxidation status of middle distance runners and swimmers [31,32]. Interestingly, a study investigating the effect of hypoxic training demonstrated that the antioxidant status in participating elite cross-country skiers was still affected at 14 days after the end of the hypoxic intervention [33]. Summarized, these examples demonstrate that acute exercise might exert an effect on oxidant/antioxidant status, whereas hypoxia might influence this status rather upon longer period training interventions. Hence, it is reasonable to assume that our acute short-term intensive exercise interventions resulted in changes of the oxidant/antioxidant status of the participating athletes. In addition, intensive exercise can alter inflammatory markers. It was demonstrated that an acute intense 3000 m time trial resulted in a differentiated inflammatory marker response. Interleukin (IL)-1β, IL-6, myeloperoxidase (MPO) and macrophage colony-stimulating factor increased, whereas IL-4 and IL-10 decreased and tumor necrosis factor α, IL-2 and IL-8 did not change [34]. Similar results were reported after a duathlon race, which caused increased IL-6 and MPO [35]. Although we did not assess oxidant/antioxidant as well as inflammatory markers in this study, we emphasize their investigation in future studies that focus on angiogenesis-related research, since these markers are involved in the control of angiogenic processes [36,37].

## 5. Conclusions

The new finding of this study is that exhausting short-term cycling exercise reveals the potential to induce ECM protein turnover resulting in a significant generation of endostatin 10 min post exercise. Thus, the presented results may contribute to a broader understanding of exercise-induced remodeling processes in the ECM and subsequently induced release and activation of angiogenic biomarkers. Both exercise scientists and medical therapists can benefit from these results in combination with discussed ones on oxidant/antioxidant as well as inflammatory markers to develop new training strategies to enhance physical capacities or new medical therapies for patients suffering from cardiovascular diseases.
